# Developing Causality and Severity Assessment Frameworks for Food Safety Signals Using Social Media Reviews: A Technical Report Based on Data From an Urban Indian Suburb

**DOI:** 10.7759/cureus.64426

**Published:** 2024-07-12

**Authors:** Akash Prabhune, Vinay Sri Hari, Neeraj Kumar Sethiya, Mansi Gauniyal

**Affiliations:** 1 Faculty of Pharmacy, School of Pharmaceutical and Populations Health Informatics (SoPPHI), DIT University, Dehradun, IND; 2 ADMIRE Centre for Advancing Digital Health, Institute of Health Management Research (IIHMR), Bangalore, IND; 3 Public Health Research, Foundation of Healthcare Technologies Society, New Delhi, IND

**Keywords:** public health monitoring, severity assessment score (sas), causality assessment index (cai), sentiment analysis, food safety surveillance, social media reviews

## Abstract

Social media reviews are a valuable data source, reflecting consumer experiences and interactions with businesses. This study leverages such data to develop a passive surveillance framework for food safety in urban India. By employing a Bidirectional Encoder Representations from Transformers (BERT)-powered Aspect-Based Sentiment Analysis tool, branded as Eat At Right Place (ERP), the study analyses over 100,000 reviews from 93 restaurants to identify and assess food safety signals. The Causality Assessment Index (CAI) and Severity Assessment Score (SAS) are introduced to systematically evaluate potential risks. The CAI uses pattern recognition and temporal relationships to establish causality while the SAS quantifies severity based on sub-aspects such as cleanliness, food handling, and unintended health outcomes. Results indicate that 40% of the restaurants had a CAI above 1, highlighting significant food safety concerns. The framework successfully prioritizes corrective actions by grading the severity of issues, demonstrating its potential for real-time food safety management. This study underscores the importance of integrating innovative data-driven approaches into public health monitoring systems and suggests future improvements in natural language processing algorithms and data source expansion. The findings pave the way for enhanced food safety surveillance and timely regulatory interventions.

## Introduction

Social media reviews have been seen as vast sources of data, presenting consumer experiences and interactions with businesses. Globally, efforts are made to utilize the data on social media for decision-making and policy-making within rapidly changing consumer industry interactions. One of the success stories in this effort is in drug safety wherein initiatives like WEB-RADR (Recognising Adverse Drug Reactions) [1] and Eudra Vigillance [2] have made successful strides in isolating specific signals of drug-linked safety reports. Food safety consumer reviews have been mainly proposed as a source of data for aiding in risk assessment [3] and supporting regulatory agencies by adding a consumer feedback loop for timely action on public health risks [4]. Developing a passive surveillance system for food safety using data available from social media has been discussed in detail by the authors of this manuscript in earlier publications [5,6]. The key missing links identified in developing a passive surveillance framework for food safety using data on social media were a) algorithm for causality assessment and b) algorithm for severity assessment.

In the area of pharmacovigilance, several casualty assessment algorithms have been developed. The most commonly used Naranjo algorithm and the World Health Organization-Uppsala Monitoring Centre (WHO-UMC) system [7] include parameters like the temporal relationship between drug administration and the onset of the adverse event; de-challenge and rechallenge outcomes, indicating improvement upon stopping the drug and recurrence upon reintroduction; the presence of alternative causes that might explain the adverse event; drug levels in the blood if measurable; previous reports of similar reactions in medical literature or databases; the response to withdrawal, showing whether the adverse event resolves after stopping the drug; and the dose-response relationship, which examines the correlation between the drug dose and the severity of the adverse event. These criteria collectively help in determining the likelihood that a drug caused or contributed to an adverse reaction.

Established severity rating scales like Common Terminology Criteria for Adverse Events (CTCAE) [8] or the Hartwig and Siegel scale [9] are used to grade the severity of drug-related adverse events. The ADR severity assessment ranges from Mild (Grade 1), where symptoms are easily tolerated and require no medical intervention, to Fatal (Grade 5), where the reaction results in death. Moderate (Grade 2) symptoms cause discomfort and minimal intervention while Severe (Grade 3) symptoms significantly interfere with daily activities and require medical intervention. Life-threatening (Grade 4) symptoms necessitate intensive medical care such as ICU admission.

The current manuscript details the process of the development of a Causality Assessment Index (CAI) and Severity Assessment Score (SAS) for food safety signals generated from consumer reviews for various restaurants.

## Technical report

The overall report is organized as the details of the methodology followed and the learnings and findings from the application of the developed CAI and SAS on data from 93 restaurants over one year. These restaurants are in the urban locality of a southern Indian city with an administrative definition of an equivalent of a suburb and a population of 90,000 to 100,000 people. To select restaurants for the study, all restaurants within the geographic boundary of the selected suburb listed on Google Maps were considered. From the total of 156 restaurants listed, those with 1000 or more reviews were deemed eligible. Consequently, 93 restaurants met this criterion and were included in the study for further analysis. The eligibility criterion of 1000 reviews was established to ensure that there was sufficient data available from restaurant reviews for the Eat At Right Place (ERP) tool to process and detect signals related to food safety elements.

Tools and software used

The authors had developed a Bidirectional Encoder Representations from Transformers (BERT)-powered Aspect-Based Sentiment Analysis tool branded as the ERP tool [5]. The ERP tool is an automated pipeline-based machine learning tool wherein it pulls review data from the Google Maps reviews section of any restaurant, the review data is pre-processed, and sentiment analysis is undertaken by the BERT model with an accuracy of 86% (F-Square value of 0.86) [10]. The ERP tool presents sentiments across seven aspects: Price, Food, Ambience, Staff, Hygiene, Variety, and Accessibility. The Hygiene aspect captures data points related to food safety like hygiene of the dining area, food handling and packaging practices, spotting of pests, or serving of undercooked/burnt food items. The ERP tool was the source of data acquisition and analysis.

We also used the Food Safety Checklist [11] used by Indian regulatory agencies for inspections of catering facilities as a guiding document to build CAI and SAS. Inspectors review the food business operator's (FBO) history and conduct on-site inspections, assessing each criterion for compliance, non-compliance, partial compliance, or non-applicability adhering to the checklist. Critical areas, marked with an asterisk (*), are prioritized due to their significant impact on food safety. Each question is scored, and total scores determine the establishment's grade: A+ (100-114) for exemplar compliance, A (91-99) for satisfactory compliance, B (77-90) for needs improvement, and no grade for scores below 77 indicating non-compliance. Establishments must address non-compliance, especially in critical areas, with immediate corrective actions.

Additionally, we used Python (Python Software Foundation, Delaware, US) for data analysis and plotting of geospatial maps.

Signal identification and distillation

The ERP tool analyzes the data into seven aspects, with hygiene being one. The list of all the restaurants registered on Google Maps in our study geography was obtained by scraping Google Maps data. Restaurants with 1000 or more reviews in Google Maps' reviews sections and operating for more than two years were included for further analysis. The selected restaurants were then subjected to analysis by the ERP tool. The reviews tagged under hygiene were further stratified and added for manual review. Two researchers manually read all the hygiene-specific reviews and classified these reviews under sub-aspects derived from the Food Safety and Standards Authority of India (FSSAI) inspection checklist.

The sub-classes included a) Cleanliness of Dining Area, b) Food-Handling Practices, c) Food-Packaging Practices, d) Pest Control, e) External Objects in Food, f) Upkeep of Infra, and g) Undercooked, Rotten, or Smelly Food. One more sub-aspect was added: h) Unintended Health Outcome Reported (e.g. Acidity, Vomiting); this was added outside the purview of the checklist since direct reporting of health outcomes can signal a higher risk of a public health emergency. Table 1 lists examples of the definitions and keywords associated with the sub-aspects.

**Table 1:** List of sub-aspects under the hygiene aspect built from the inspection checklist of an Indian regulatory agency

Sub-aspect	Short Definition	Keyword
Cleanliness of the Dining Area	Defined as the cleanliness of the dining area, including the sitting area and entry and exit points	Unclean tables, dirty floors
Food-Handling Practices	Defined as the practices of handling food and personal hygiene followed while carrying and serving the food by the service team	Dipped hands in drinks, the waiter looked unhealthy
Food-Packaging Practices	Defined as the practices associated with the storage and transport of prepared food and raw materials	Wrapped food in old newspaper, the container was leaking
Pest Control	Defined as the reports associated with spotting pests in the premises and finding pests in the served food	Saw a rat in the washroom, found a fly in the soup
External Objects in Food	Defined as the reports associated with spotting non-edible items in the served food	Stone in the rice, hair in the curry
Upkeep of the Infra	Defined as the overall upkeep and maintenance of the establishment, including lightning, ventilation, dampening of the walls and ceiling, toilets, and washrooms.	Poorly ventilated, AC not working
Undercooked, Rotten or Smelly Food	Defined as the reports over the quality of the food served	Chicken was undercooked, cheese was smelly, bread had fungus developed
Unintended Health Outcome Reported (e.g. Acidity, Vomiting)	Defined as the voluntary reporting of any health outcome like vomiting, acidity, loose motion, or headache by the consumer	Vomited after eating mayonnaise, had stomach ache due to biryani

These sub-aspects were manually identified for each review under the aspect of Hygiene. Also, the sub-aspects were carrying positive or negative labeling provided by the ERP tool; for example:

“The chicken sandwich was delicious with crispy sourdough bread and juicy chicken inside. The bread tasted fresh and the sourness was accentuating the Flavors. Served with crispy potato wedges and a coleslaw, the whole meal is quite filling.”

This review was marked as positive under hygiene sentiment by the ERP tool due to keywords like Fresh, Crispy, and Juicy; the researchers marked anti against the sub-aspect of undercooked, rotten, or smelly food. On the contrary, the following review:

“Mocha caramel cake slice and the cream doughnut were sooo hard that they seemed to have been made at least a week ago.”

was identified by the ERP tool and the researchers re-labeled it as negative under the sub-aspect of Undercooked, Rotten, or Smelly Food. This exercise was carried out on 1005 hygiene aspects identified reviews by ERP tool from 93 restaurants, with a total of 100,614 reviews processed. To maintain the privacy of the restaurants, the restaurants were broadly categorized into Casual Dining Restaurants (n = 63), Fine Dining Restaurants (n = 2), Buffet Restaurants (n = 4), Quick Service Restaurants (n = 13), and Food Truck and Dhaba (n = 11). This sub-aspect classification helped in detecting the signal aligning with the food safety checklist by the regulatory agency.

Development causality assessment index (CAI)

The WHO-UMC uses the temporal relationship between drug administration and the onset of the adverse event: de-challenge and rechallenge to assess the causality, which requires prospective interactions with subjects. This is not feasible for passive systems based on secondary data. The WEB-RADR researchers have used Pattern Recognition and Temporal Relationships to establish causal relationships. Similarly, the current research uses the Pattern Recognition and Temporal relationship to build the CAI. For pattern recognition, the ERP tool lists all the positive and negative sentiment reviews for a particular restaurant, it checks for two key patterns - a) Lexical Diversity and b) Similarity Index - designed to avoid duplication of data and prove the reviews are written by the same individual. Table 2 highlights the Pattern Recognition done by the ERP tool.

**Table 2:** Illustration of pattern recognition carried out for CAI Both sentences exhibit high lexical diversity (TTR of 0.61 and 0.53) but have a low Jaccard similarity (0.16) and no common bigrams. This indicates distinct vocabulary and phrase usage, suggesting that the sentences were likely written by different individuals. CAI: Causality Assessment Index; TTR: Type-Token Ratio

Metric	Sentence 1	Sentence 2	Comparison/Comment
Sentence	"It was my first time to taste this dish and totally satisfied. I cut down that 5th star because it lacks spice and realized that there are spicy sauces that can be added to the dish, after our plates were empty. Good ambience and service."	"Whoever people looking for delicious food and humble service at the place of absolute sizzler only i can say that I had good experience there especially i heard about that discount on ala carte menu and on the buffet 50off that was really cool and great dine to have, at that reasonable price number of buffet items which were making us feel tasty thanks to absolute Sizzlers ...."	
Word Count	36 words	95 words	Sentence 2 is significantly longer than Sentence 1.
Unique Words	22 unique words	50 unique words	Sentence 2 has more unique words due to its length.
Type-Token Ratio	22/36 ≈ 0.61	50/95 ≈ 0.53	Both sentences have a relatively high TTR, indicating diverse word usage, but Sentence 1 has a slightly higher TTR.
Jaccard Similarity	10 (common words) / 62 (total unique words) ≈ 0.16		Low Jaccard Similarity suggests limited overlap in vocabulary between the two sentences.
Common Words	"I", "that", "the", "to", "and", "service", "good", "experience", "dish", "food"		Only 10 common words out of 62 unique words across both sentences.
Common Bigrams	"first time", "taste this", "totally satisfied"	"good experience", "buffet 50off", "absolute sizzler"	No common bigrams between the two sentences.
Bigram Overlap	0	0	The lack of common bigrams indicates different phrase usage patterns.

The temporal relationship analysis was focused on highlighting the event proximity to eliminate the chance. We conducted a chi-square analysis to test for the temporality of events being due to underlying issues at the restaurant and not due to chance. For example, Table 3 plots the number of hygiene-related reviews highlighted by the ERP tool for a casual dining restaurant. A chi-square test was conducted for total reviews and hygiene aspect negative reviews yielding a chi-square value of 35.80 and a P value of 2.08. The chi-square for total reviews and hygiene aspect positive reviews yielded a chi-square value of 5.01 and a P value of 0.6, indicating the spike in negative reviews in June was due to chance.

**Table 3:** Illustration of temporal assessment data of a restaurant for estimation of CAI CAI: Causality Assessment Index

Month	Total Reviews	Hygiene Aspect Positive Review	Hygiene Aspect Negative Reviews	Proportion of Negative Reviews to Total Reviews
January	50	4	5	10%
February	40	7	4	10%
March	60	6	6	10%
April	45	8	4	8.9%
May	55	5	5	9.1%
June	30	3	15	50%
July	50	4	5	10%
August	40	3	4	10%

The pattern recognition and temporal assessment for chance was conducted for the data of all 93 restaurants and the restaurants where patterns indicated data was duplicated or the review spikes were due to chance were excluded as noise. The restaurant's data qualifying patterns recognition and temporal assessment were included for CAI. The index was formulated as

$CAI=\frac{Number\ of\ negative\ hygiene\ aspect\ reviews}{Number\ of\ positive\ hyigene\ aspect\ reviews}$

In cases where negative hygiene aspect reviews were higher than positive hygiene aspect reviews, the CAI would be higher than 1, indicating that more signals are generated from reviews over the hygiene aspect of the restaurant and there is some genuine concern that needs regulatory intervention.

Development of Severity Assessment Score

Upon establishment of causality (restaurants with CAI <1), the next step was to assess severity. To assess the severity number of negative hygiene aspects reviews were stratified according to sub-aspects defined in Table 1. The weights proportional to the weightage for sub-aspects assigned in the Food Safety Checklist were assigned as follows: Cleanliness of Dining Area: 2 points per negative review in the stipulated duration, External Objects in Food: 3 points per negative review in the stipulated duration, Food Handling Practices: 2 points per negative review in the stipulated duration, Food Packaging Practices: 1 point per negative review in the stipulated duration, Poor Pest Control: 3 points per negative review in the stipulated duration, Undercooked, Rotten or Smelly Food: 3 points per negative review in the stipulated duration, Upkeep of the Infrastructure: 2 points per negative review in the stipulated duration, and Unintended Health Outcomes (Acidity, Vomiting, Loose Motion): 4 points per negative review in the stipulated duration. If a restaurant receives 1 negative review under sub-aspect the SAS score would be 20 points. We graded the severity as a score of less than 5 was a low risk to public health, a score between 6 to 10 was a slightly higher risk to public health, a score between 11 to 15 was a high risk to public health, a score between 16 to 20 was a higher risk to public health and score above 20 was the highest risk to public health. The formula for SAS estimation is explained below.

$SAS=\sum\left(Numberof\ Negative\ Category\times W e i g h t o f t h e C a t e g o r y\right)\mathrm{Risk\ Score}$

Table 4 illustrates the SAS calculation for a Casual Dining Restaurant, Buffet Restaurant, Quick Service Restaurant, and Food Truck or Dhaba.

**Table 4:** Illustration of SAS estimation for a restaurant of each category with a CAI score above one CAI: Causality Assessment Index; SAS: Severity Assessment Score

Categorization	Buffet	Casual Dining	QSR	Food Truck/Dhaba
Number of Negative Hygiene Aspect Review	6	6	3	6
Number of Positive Hygiene Aspect Review	2	4	1	3
Causality Assessment Index (CAI)	3.00	1.50	3.00	2.00
Cleanliness of the Dining Area	3	0	0	1
External Objects in Food	0	0	1	0
Food-Handling Practices	1	1	0	0
Food-Packaging Practices	0	0	1	0
Poor Pest Control	0	0	0	0
Undercooked, Rotten or Smelly Food	2	5	0	5
Unintended Health Outcomes (Acidity, Vomiting, Loose Motion)	0	0	0	0
Upkeep of the Infra	0	0	1	0
Severity Assessment Score (SAS)	11	17	5	16
Category of SAS	High Risks To Public Health	Higher Risk To Public Health	Low Risk To Public Health	Higher Risk To Public Health

Learnings from the application of the CAS and SAS to the data of 93 restaurants

**Figure 1:**
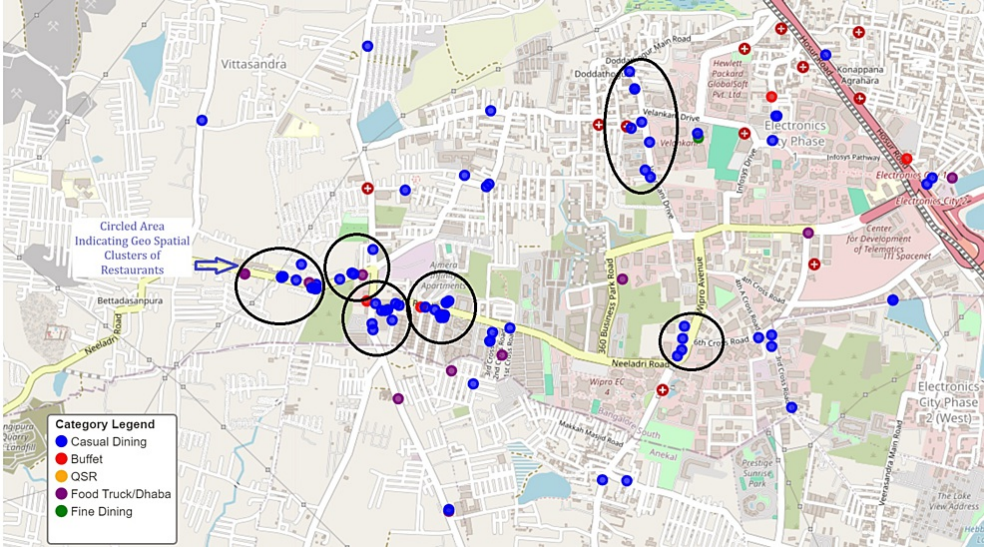
Description of the area with labeled restaurants as per categories A distortion code was used while plotting the restaurants on the GIS. To avoid exact identification of the restaurants, the distortion code randomly changed latitude and longitude by 5 meters to 10 meters. GIS: geographic information system

The locality selected is a mix of residential areas and office spaces with a highway (Hosur Road) on the right side of the locality. A main road named Neeladri Road is a commercial road with major residential complexes opening on the road followed by ancillary roads like Velannkani Drive and Wipro Avenue joining on the main road. Figure 1 depicts the geospatial mapping of the restaurants according to the categories of Casual Dining, Fine Dining, Buffet, Quick Service Restaurant, and Food Truck or Dhaba.

Of the total 93 restaurants included in the study, 68% were Casual Dining Restaurants, 14% were Quick Service Restaurants, 12% were Food Trucks/Dhabas, 4% were Buffets, and 2% were Fine Dining Restaurants. Table 5 presents the description of data. 2 restaurants from the Casual Dining category had issues with patterns of data temporal assessment and thus were not assessed for CAI.

**Table 5:** Description of the restaurants including category-wise CAI and SAS averages * For the fine-dining restaurants, no negative sentiment reviews were detected by the ERP tool, thus CIA analysis was not applicable for the two fine-dining restaurants. CAI: Causality Assessment Index; SAS: Severity Assessment Score

Restaurant Category	Number of Restaurants	No. of Restaurants Review data qualified for CAI after Pattern and Temporal Assessment	No. of Restaurants with CAI above 1	Average CAI score for Category	Average SAS for the category
Fine Dining	2	2	0	0.2	NA*
Casual Dining	63	61	22	1.26	13.5
Quick Service Restaurant	13	13	4	5	10.75
Buffet	4	4	2	2.5	16.5
Food Truck/Dhaba	11	11	6	3.7	12.1

Forty percent of the restaurants across categories had CAI scores above 1, indicating that more of these restaurants had more negative reviews for the hygiene aspect than positive reviews for the hygiene aspect thus establishing causality. Table 6 presents the number of reviews classified as positive and negative for hygiene aspects and the sub-aspect classification of negative labeled review instances used for SAS.

**Table 6:** Hygiene aspect reviews and sub-aspect classifications for different types of dining establishments

Category	Fine Dining	Casual Dining	Quick Service Restaurants	Buffet	Food Truck/Dhaba
Hygiene Aspect Classified by the ERP Tool					
No. Negative Labelled Hygiene Reviews	4	311	61	16	67
No. Positive Labelled Hygiene Reviews	20	437	92	16	65
Sub-Aspect Classification Manual by Researchers (only negatively labeled reviews from the Hygiene aspect of the ERP tool are manually classified, positive reviews are not classified)					
Cleanliness of the Dining Area	0	62	21	3	20
External Objects in Food	0	34	6	6	5
Food Handling Practices	2	57	6	2	12
Food Packaging Practices	1	18	8	1	0
Poor Pest Control	0	27	3	0	9
Undercooked, Rotten, or Smelly Food	1	82	13	4	14
Unintended Health Outcomes (Acidity, Vomiting)	0	8	0	0	0
Upkeep of the Infra	0	23	4	0	7

Fine Dining restaurants had no casual linkage since the number of positive reviews was higher than negative reviews of the Hygiene aspect for both restaurants. Casual Dining restaurants were the only category wherein unintended health outcomes like vomiting, acidity, or stomach aches were reported. Cleanliness of the dining area sub-aspect had the highest number of negative reviews across categories. Chicken was the most common food item to be reported undercooked while paneer, cheese, and fish were food items reported to be rotten. Bread and cake were food items reported to be served stale.

**Figure 2:**
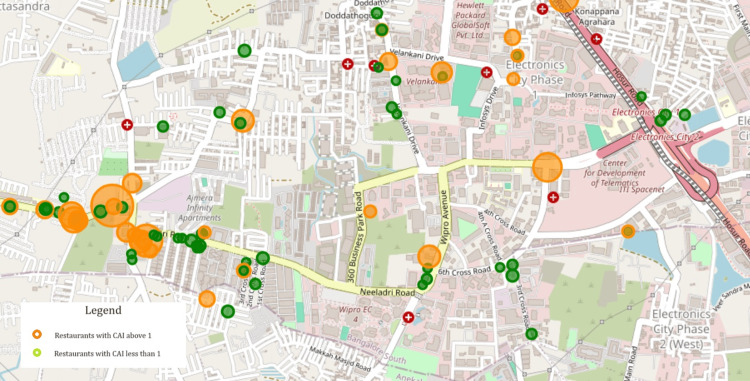
Geo-spatial mapping of the restaurants with CAI and SAS CAI: Causality Assessment Index; SAS: Severity Assessment Score

In terms of SAS score, 3 restaurants had a score above 20 indicating the highest risk for public health, 2 being from the Casual Dining Category and 1 from the Buffet category. Figure 2 presents a geospatial map of restaurants, with green indicating restaurants with a CAI less than 1 and orange representing restaurants with a CAI above 1. The bubble size for the orange color increases with higher SAS.

The CAI and SAS have yielded intriguing results, and the results generated can pinpoint particular establishments with a high risk of food safety leading to public health issues. The graded SAS also enables the prioritization of corrective actions.

## Discussion

Learnings from literature

Studies have shown that consumer reviews on platforms like Yelp and Google Maps can be harnessed to identify potential food safety issues. For example, Harrison et al. demonstrated that online reviews could be a valuable tool for foodborne illness surveillance [12]; however, the work relied on using machine learning to identify food-safety-related tweets and to ask them to report to the health department for further action. This approach had major collaboration between the research team and health department and reliance on the consumer to report the events to authorities; this approach works with limited success in developing countries like India where appropriate channels for reporting food safety issues are not available, and in case complaints are registered, regulators seldom respond to the complaints. Similarly, a paper by Nsoesie et al. has presented a working model for signal detection and prediction of possible outbreaks geo-spatially using data from social media [13]. However, the approach was sending all the user-reported food safety data identified through a machine learning sentiment analysis to regulators. This adds data with a lot of noise and hinders regulators' actions. A paper by Michael Siering used machine learning-based classifiers to predict the health violations of restaurants based on online reviews [14]. The approach by Siering includes information on reports from previous health inspections and accounts for factors like restaurant popularity along with the linguistic and textual composition of reviews. This approach, though different from ours, focuses on highlighting the risk of foodborne illnesses. In the context of developing countries, data on food inspection is rarely available to the public, thus the current approach relies on consumer review data to detect public health risks is deemed practical. 

Challenges and limitations

The sentiment analysis performed by the ERP tool, although highly accurate, is still subject to the nuances of language and context, which can lead to misclassification. Additionally, the manual review process, while thorough, is time-consuming and prone to human error. The reliance on data from Google Maps also introduces a selection bias, as not all consumers leave reviews, and those who do may not represent the broader population.

Implications of this work

The development of the Causality Assessment Index (CAI) and Severity Assessment Score (SAS) provides a structured approach to evaluating food safety signals from social media reviews. This methodology offers a systematic way to identify and prioritize potential food safety issues, enabling regulatory agencies to take timely and targeted action. By incorporating both pattern recognition and temporal assessment, the CAI ensures that only genuine signals are considered, reducing the noise from spurious reviews. The SAS further refines this by quantifying the severity of the identified issues, allowing for a risk-based approach to food safety management. This framework can be adapted and applied in other contexts and geographies, enhancing the overall food safety surveillance infrastructure.

Future direction

Future research should focus on improving the automation and accuracy of the ERP tool. Enhancing natural language processing algorithms to better understand context and reduce misclassification will be crucial. Additionally, expanding the data sources beyond Google Maps to include other review platforms and social media sites can provide a more comprehensive picture of food safety. There is also a need for longitudinal studies to validate the effectiveness of the CAI and SAS over time and across different regions. Collaborations with regulatory agencies and food businesses can facilitate the integration of this framework into existing food safety monitoring systems, driving broader adoption and impact.

## Conclusions

The development and application of the Causality Assessment Index (CAI) and Severity Assessment Score (SAS) for food safety signals derived from social media reviews offer a novel and effective approach to food safety surveillance. By leveraging advanced machine learning techniques and structured analysis frameworks, this study demonstrates the potential of using consumer-generated data to identify and prioritize food safety risks in real time. The findings indicate that social media reviews can provide valuable insights into hygiene practices and potential health hazards in food establishments, enabling timely and targeted regulatory interventions. While challenges such as data variability and manual review processes remain, the structured methodology proposed here lays the groundwork for future enhancements and broader implementation. Continued refinement of natural language processing algorithms and expansion of data sources will further enhance the accuracy and applicability of this framework. Ultimately, this work underscores the importance of integrating innovative data-driven approaches into public health monitoring systems, paving the way for more proactive and responsive food safety management.
